# Iatrogenic Pulmonary Nodule in a Heart Transplant Recipient

**DOI:** 10.1155/2014/546209

**Published:** 2014-10-27

**Authors:** Atul C. Mehta, Juan Wang, Jarmanjeet Singh, Joseph Cicenia

**Affiliations:** ^1^Department of Pulmonary Medicine, Respiratory Institute, Cleveland Clinic, Cleveland, OH 44195, USA; ^2^Department of Pulmonary Diseases, Tiantan Hospital, Beijing 100050, China; ^3^Maharaja Agrasen Medical College, Agroha 125047, India

## Abstract

A 58-year-old female with a history of non-Hodgkin lymphoma and end-stage nonischemic cardiomyopathy from Adriamycin toxicity underwent orthotic heart transplantation during June 2013. She developed shortness of breath in September 2013 and was suspected to have invasive pulmonary aspergillosis. A flexible bronchoscopy (FB) with a transbronchial biopsy (TBBx) was performed. She was found to have a focal lung nodule in the same location at the site of the TBBx on day 13 after the FB. Spontaneous resolution of the nodule was confirmed on the computed tomography (CT) scan of chest performed at 3 months. We believe that this nodule was as a consequence of the TBBx. Formation of a peripheral pulmonary nodule (PPN) following a TBBx is occasionally encountered among the recipients of the lung transplantation. To our knowledge, this is the first case of TBBx producing a pulmonary nodule in a heart transplant recipient. Physicians caring for the patients with heart transplantation should be cognizant of the iatrogenic nature of such nodule to avoid unnecessary diagnostic work-up.

## 1. Introduction

Peripheral pulmonary nodule (PPN) is a common clinical challenge for the pulmonologists given a wide range of its differential diagnosis. When present in the recipients of solid organ transplantation, these nodules represent even a greater challenge due to the possibilities of an opportunistic infection, pulmonary infarcts, posttransplant lymphoproliferative disorder (PTLD), and malignancies [1]. Prompt evaluation and appropriate treatment of the PPN are essential in this high risk population.

Recently, we noticed a transient appearance of PPNs in a heart transplant recipient who underwent FB with a TBBx to rule out an opportunistic infection. To our knowledge, appearance of a PPN following a TBBx in a heart transplant recipient has never been reported. Based on its temporal relationship with the procedure, the nodule in our patient was thought to be related to the TBBx. The nodule caused no symptoms; hence, no diagnostic work-up was undertaken and it resolved spontaneously. We believe that this is the first case of iatrogenic PPN appearing in a heart transplant recipient as a result of the TBBx. The physicians caring for the heart transplant recipients should be aware of this phenomenon to avoid unnecessary diagnostic work-up.

## 2. Case Report

A 58-year-old female was diagnosed with non-Hodgkin lymphoma in 1996 and was treated successfully with chemotherapy. She developed end-stage nonischemic cardiomyopathy from Adriamycin toxicity and underwent orthotic heart transplantation during June 2013. Three months into her transplantation, she developed shortness of breath and was found to have nonspecific pulmonary infiltrates and without any evidence of a pulmonary nodule on a CT of the chest. She was suspected to have invasive pulmonary aspergillosis (IAP) and was placed on oral voriconazole (400 mg bid). A flexible bronchoscopy (FB) was performed in a usual fashion under conscious sedation. Bronchoalveolar lavage (BAL) and five TBBx specimens were obtained from the right lower lobe (RLL) anterior segment under fluoroscopic guidance. There were no complications and the amount of bleeding was minimal. The histological examination of the biopsy revealed alveolated lung parenchyma with no pathologic diagnosis. The special stains for fungal organisms were negative.

**Figure 1:**
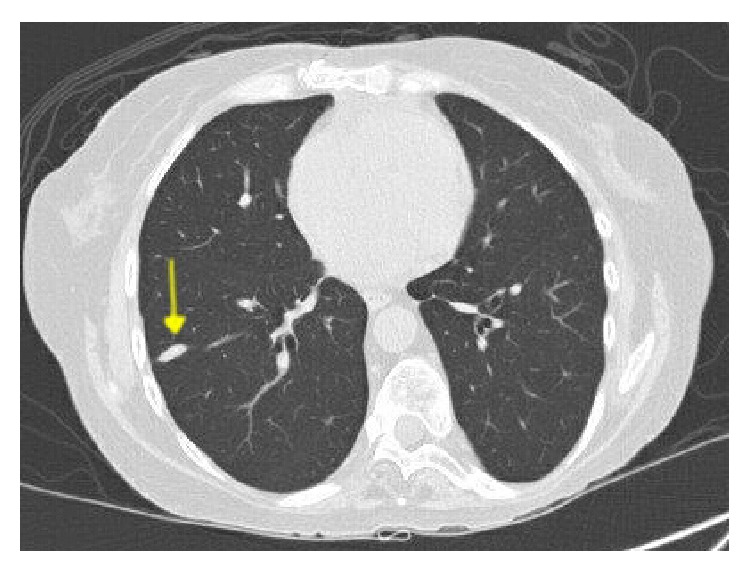
Computed tomography scan of the chest revealing a 19 × 8 mm nodule (arrow) involving the area of transbronchial biopsy on day 20 of the procedure.

**Figure 2:**
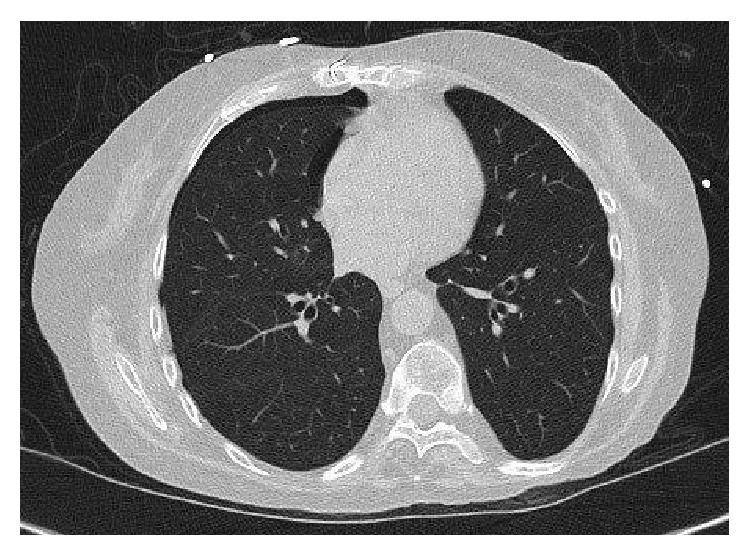
Computed tomography scan of the chest revealing resolution of nodule at 3 months.

A repeat chest X-ray on day 5 of the FB revealed a subtle opacity overlying the RLL in close proximity to oblique fissure. On day 13 of the procedure, a 9 mm RLL pulmonary nodule was detected on a repeat chest film. CT scan of the chest a week later showed a focal oval-cylindrical opacity at the peripheral aspect of RLL measuring 19 × 8 mm. Interestingly, the location of the opacity matched the site of TBBx. Besides, it showed resolution of the nonspecific pulmonary infiltrates (Figure 1). The patient continued to show subjective improvement on empiric voriconazole therapy. In view of patient's stable pulmonary status, this nodule was thought to be related to the TBBx and no further diagnostic work-up was carried out. A repeat CT scan a month later, 7 weeks following the procedure, was unremarkable, except for the RLL PPN, which reduced to 16 × 6 mm in size. A follow-up CT scan of the chest at 3 months from the FB revealed total resolution of the pulmonary nodule (Figure 2) [2].

## 3. Discussion

It is a conservative estimate that over 200,000 pulmonary nodules will be detected in year 2014 in the United States, outside of the lung cancer screening program [3, 4]. Peripheral pulmonary nodules (PPN) are a common radiographic finding and are still considered a clinical dilemma. The appearance of this nodule among the recipients of solid organ transplantation is of added significance as it includes differential diagnosis such as PTLD (39%), invasive pulmonary aspergillosis (IAP) (37%), pulmonary embolism (PE), and other opportunistic infections (5–9%) [1].

The common causes of pulmonary nodules undergoing spontaneous resolution include infections, PE, pulmonary pseudotumor, waxing, and waning bronchoalveolar adenocarcinomas. We do not believe that this nodule was caused by any of the above. First of all, if it was due to a fungal infection, it would not have appeared in the patient already undergoing treatment with voriconazole. Second, it was supposed to show a decrease in the size rather than enlargement in post-TBBx period especially when the nonspecific pulmonary infiltrates resolved with voriconazole treatment. Third, BAL culture and TBBx specimens for fungal organisms were negative. Fourth, it resolved without addition of any newer antifungal drug. The timing (after TBBx) and location (at the site of biopsy) of the PPN make other causes like PE, pseudotumor, and bronchoalveolar carcinoma very unlikely.

Our patient, a heart transplant recipient, developed a PPN nodule following a TBBx. Although Root et al. showed a frequent occurrence of these nodules in lung transplant recipients (35%), to the best of our knowledge, it has never been reported in the heart transplant patient population. We also agree with Root et al. that this phenomenon is most likely due to a local hematoma and an impaired lymphatic drainage of the lung due to the major thoracic surgery [5]. We speculate that size of the nodule may depend upon a number of samples obtained from a single location. As observed, this nodule could appear as early as within a week after the procedure and may take 3 months to resolve. Given the fact that it resolved spontaneously, that is, without addition of any new treatment, its diagnosis and management require only a good temporal relationship and close follow-up.

## 4. Conclusion

In heart transplant recipients, transbronchial lung biopsy can cause a PPN. It appears in the same segment as that of the procedure. Physicians involved in caring for the heart transplant recipients should be cognizant of this newer iatrogenic etiology of a PPN. The awareness regarding this finding will avoid unnecessary work-up in this unique group of patients.

## Abbreviations

TBBx: Transbronchial lung biopsy
PPN: Peripheral pulmonary nodule
PTLD: Posttransplant lymphoproliferative disorder
IAP: Invasive pulmonary aspergillosis
RLL: Right lower lobe of the lung
FB: Flexible bronchoscopy
BAL: Bronchoalveolar lavage.
